# Cultural heritage education and civic engagement: a value-socialization model among Chinese university students

**DOI:** 10.3389/fpsyg.2026.1779128

**Published:** 2026-02-26

**Authors:** Xu Yu

**Affiliations:** School of Animation and Games, Hangzhou Polytechnic University, Hangzhou, Zhejiang, China

**Keywords:** civic engagement, cultural heritage education, cultural identity, cultural pride, educational socialization, higher education, structural equation modeling, student development

## Abstract

Cultural heritage education (CHE) has been increasingly promoted in higher education as a means of fostering students' civic engagement, yet the psychological mechanisms through which such educational experiences translate into civic outcomes remain insufficiently specified. Drawing on value socialization perspectives, the present study proposes and tests a mechanism-oriented model in which CHE influences civic engagement through two complementary internalization pathways: cultural identity, conceptualized as a cognitive process of self-definition, and cultural pride, conceptualized as an affective evaluative orientation toward cultural heritage. Using survey data from 744 Chinese undergraduate students, structural equation modeling was employed to compare a fully mediated model with a partial-mediation specification that allowed for residual direct effects of CHE on civic engagement. Results indicated that CHE was positively associated with both cultural identity and cultural pride, which independently predicted civic engagement, and bootstrap analyses confirmed significant indirect effects through both pathways, while a residual direct association between CHE and civic engagement remained. These findings advance existing research by operationalizing value socialization as a dual-process mechanism involving parallel cognitive and affective pathways, clarifying how heritage education may foster civic engagement through both meaning internalization and positive emotional evaluation. The study contributes to theoretical refinement of value socialization models and offers practical implications for the design of cultural heritage education in higher education, particularly in the Chinese context.

## 1 Introduction

Higher education is widely recognized as a critical socialization context for young adults, in which students' values, identities, and orientations toward public life continue to develop alongside academic learning. Beyond disciplinary knowledge acquisition, universities play an important role in shaping students' civic-related attitudes and engagement by exposing them to shared cultural meanings, collective narratives, and opportunities for participation. Within this educational psychology perspective, learning experiences embedded in the curriculum and campus environment can function as formative contexts that influence how students interpret their social responsibilities and their relationship to the broader community.

Cultural heritage education has increasingly become a salient component of higher education in China, reflecting broader societal efforts to strengthen cultural confidence and to reconnect tradition with contemporary public life. Beyond the transmission of specialized knowledge, universities are widely recognized as strategic settings for cultivating young adults' value orientations, identity development, and public-minded behaviors. Within this broader educational mission, cultural heritage education—implemented through curriculum design, campus cultural initiatives, and experiential learning—has been regarded as a meaningful pathway for fostering students' civic engagement. Prior work in cultural education and civic development suggests that when students encounter heritage-related knowledge and practices in structured educational settings, they may develop stronger cultural attachment and interpret heritage as relevant to contemporary community issues, which can be associated with higher willingness to participate in civic and community affairs (Liu et al., 2023; Zhao and Lee, 2024; Felices-De la Fuente et al., 2020).

From a value-socialization perspective, educational institutions function as crucial agents through which culturally endorsed norms, values, and narratives are transmitted to younger generations. Importantly, value socialization does not merely involve the acquisition of cultural knowledge; it also entails the internalization of beliefs about collective belonging and prosocial obligations (Grusec and Goodnow, 1994; Benish-Weisman et al., 2013). For university students, cultural heritage education may therefore operate as a comprehensive socialization context that legitimizes cultural values, integrates personal development with national and community narratives, and provides symbolic resources for interpreting what it means to be a constructive member of society (Liu et al., 2025; Sagiv et al., 2022; Pascarella and Terenzini, 1991). In parallel, higher education research emphasizes that civic engagement among emerging adults is shaped not only by civic knowledge and skills but also by identity- and emotion-based commitments that sustain participation (Ehrlich, 2000; Zaff et al., 2010; Flanagan and Levine, 2010; Finlay et al., 2010).

Cultural heritage education is commonly conceptualized as an educational practice that integrates tangible and intangible cultural resources into learning contexts, with the aim of enhancing learners' cultural understanding, appreciation, and awareness of cultural continuity (Valencia Arnica et al., 2023; Monteagudo-Fernández et al., 2021). Within university settings, heritage-related courses and educational activities may enable students to perceive cultural traditions not as static historical artifacts but as living resources that remain relevant to contemporary social issues and collective wellbeing (Sweeney and Tanaka, 2022). Empirical evidence further indicates that cultural learning experiences are associated with heightened community awareness, stronger endorsement of shared civic norms, and an increased willingness among young people to contribute to public life (Cuenca-López et al., 2021; Chaparro-Sainz et al., 2022). Together, these findings suggest that cultural heritage education may be meaningfully linked to civic engagement, insofar as it strengthens students' sense of meaning, social embeddedness, and the perceived relevance of civic participation in everyday life.

However, the association between cultural heritage education and civic outcomes is likely to be better understood by specifying the psychological mechanisms through which heritage-related learning is internalized. Two constructs that capture this internalization are cultural identity and cultural pride. Cultural identity refers to individuals' cognitive recognition of belonging to a cultural group and their endorsement of shared symbolic meanings and values (Phinney, 2013; Ashmore et al., 2004; Umaña-Taylor et al., 2014; Juang and Syed, 2010). From a social identity perspective, when group membership becomes salient and meaningful, individuals are more likely to internalize group-relevant norms and develop commitments to collective welfare (Tajfel et al., 2001; Van Zomeren et al., 2008; Tajfel and Turner, 2004). In higher education contexts, heritage learning may heighten students' awareness of cultural membership and strengthen their connection to culturally embedded narratives, thereby consolidating cultural identity (Santana et al., 2024; Mathews et al., 2022). As a result, students with a stronger cultural identity may be more inclined to endorse civic norms and to view civic participation as consistent with their self-concept and group-related expectations.

Complementing the cognitive dimension, cultural pride reflects positive emotional attachment and evaluative affirmation toward one's cultural heritage (Smith et al., 2007). Beyond cognitive identification, affective processes can play a distinct and complementary role in linking cultural learning to civic outcomes. According to broaden-and-build perspectives on positive emotions, pride can expand individuals' value horizons, strengthen social bonds, and foster prosocial orientations that extend beyond the self (Fredrickson, 2001). In educational settings, heritage-related learning experiences that highlight cultural resilience, creativity, and historical continuity may elicit cultural pride, thereby motivating students to contribute to the vitality and well-being of their cultural communities (Leach et al., 2007). Heritage-related experiences—such as campus exhibitions, heritage festivals, cultural practice workshops, or community-based cultural projects—may further evoke admiration and pride, increasing students' willingness to support public life and collective flourishing.

Taken together, these lines of reasoning suggest that cultural heritage education may be associated with civic engagement through complementary cognitive and affective routes. Specifically, heritage-oriented learning can strengthen students' cultural identity and cultural pride, and these orientations may, in turn, be linked to higher civic engagement. At the same time, heritage education may also relate to civic engagement through additional pathways not explicitly modeled here (e.g., increased exposure to civic-relevant norms, enhanced civic efficacy, or greater opportunities for participation), making it prudent to evaluate both fully mediated and partial-mediation specifications when testing the model (Maxwell and Cole, 2007; Cole and Maxwell, 2003).

Despite growing scholarly interest in the intersections between cultural education and civic engagement, several gaps remain. First, prior research has often examined cultural identity, cultural pride, and civic engagement in isolation, with fewer studies integrating these constructs into a unified framework that clarifies their joint roles in value-socialization processes (Valencia Arnica et al., 2023). Second, although cultural heritage education has been increasingly discussed as a potential driver of civic development, empirical work that explicitly tests both cognitive and affective cultural mechanisms as concurrent routes to civic engagement remains limited, particularly among university student populations (Yan and Li, 2023). Third, in the Chinese context, higher education policies and practices have placed growing emphasis on leveraging traditional culture for youth development; however, model-based evidence is still needed to demonstrate how heritage education translates into civic engagement under contemporary campus conditions (Liu et al., 2023).

**Figure 1:**
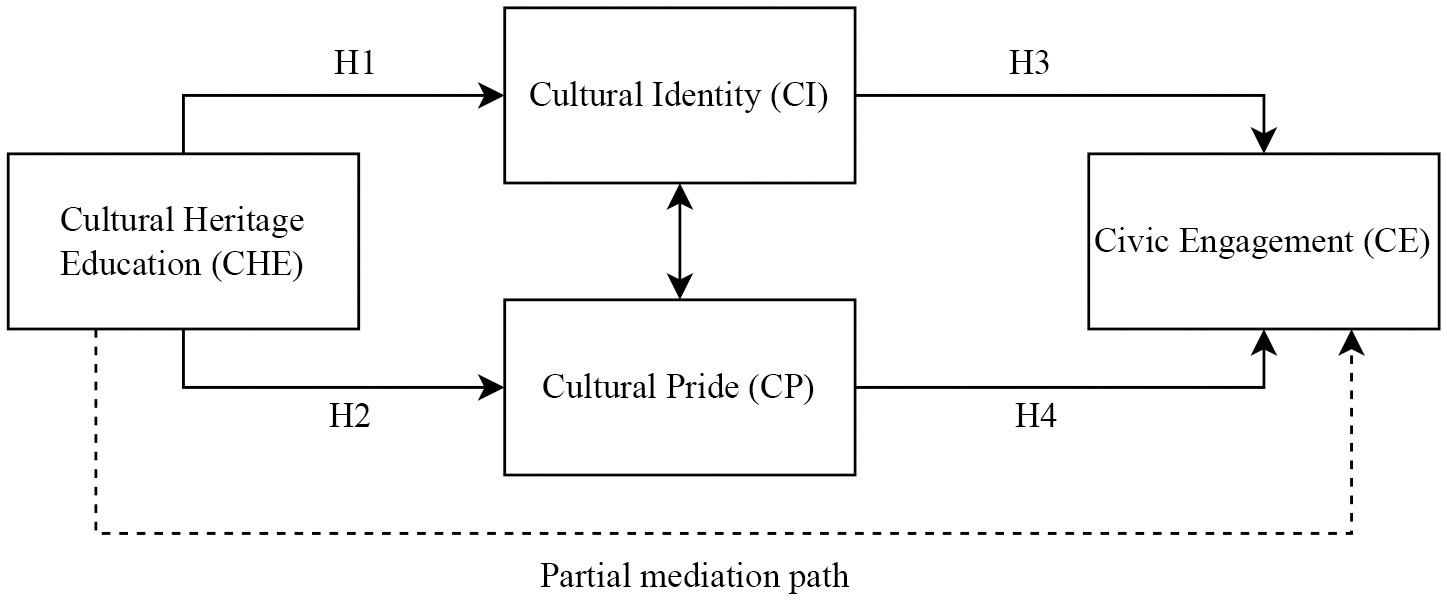
Proposed value-socialization model. Cultural heritage education (CHE) is expected to relate to civic engagement (CE) through two parallel psychological mechanisms: cultural identity (CI) and cultural pride (CP). A residual direct path from CHE to CE is additionally examined to test partial mediation and to benchmark the fully mediated baseline model.

To address these gaps, the present study proposes and tests a value-socialization model among Chinese university students in which cultural heritage education (CHE) functions as the antecedent, cultural identity (CI) and cultural pride (CP) serve as complementary internalization mechanisms, and civic engagement (CE) represents the outcome. The model emphasizes parallel mediation through CI and CP while also allowing for a residual direct association between CHE and CE to reflect additional unmeasured pathways. By integrating these constructs into a single explanatory framework and comparing a partial-mediation specification with a fully mediated baseline, this study aims to provide a more theoretically precise and methodologically rigorous account of how cultural heritage education relates to civic engagement. Based on this conceptual framework, the proposed model is presented in Figure 1, and the hypotheses are formulated below.

Building on value socialization perspectives in education and civic development (Grusec and Goodnow, 1994), the present study does not aim to introduce value socialization as a novel theoretical concept. Rather, it seeks to operationalize value socialization processes within the specific context of cultural heritage education in higher education. In doing so, this study advances prior work by specifying two parallel psychological pathways—cognitive identification and affective evaluation—through which heritage education may translate into civic engagement outcomes.

## 2 Theoretical model and hypotheses development

To integrate relevant theory and prior empirical insights, this study advances a value-socialization model linking cultural heritage education (CHE) to civic engagement (CE) among university students. Value-socialization theory emphasizes that educational contexts are associated with civic-related behavior by fostering internalized orientations and values that can guide individuals' public-minded action. In the present context, cultural heritage education is conceptualized as a salient socialization environment in higher education that may be associated with both cognitive and affective orientations toward heritage, which are in turn related to civic engagement.

### 2.1 Conceptual model overview

The proposed model specifies two complementary psychological mechanisms through which CHE relates to CE: a cognitive pathway represented by cultural identity (CI) and an affective pathway represented by cultural pride (CP). Rather than assuming a strict sequential process, CI and CP are theorized to operate in parallel. That is, heritage-related learning experiences may simultaneously strengthen students' sense of cultural self-definition (identity) and their positive emotional attachment to heritage (pride), and both orientations can motivate civic participation.

At the same time, the model allows for the possibility that cultural heritage education may also be associated with civic engagement through additional unmeasured mechanisms (e.g., civic efficacy, perceived social norms, or opportunities for participation). Therefore, in addition to the hypothesized indirect pathways, an alternative structural specification (partial mediation) that includes a direct CHE → CE path is examined empirically to provide a more realistic and reviewer-robust account of the relationship (see Section 4 for model comparison).

#### 2.1.1 Cultural heritage education and cultural identity

Cultural identity reflects individuals' cognitive recognition of cultural membership and the internalization of shared symbols, meanings, and narratives. Universities provide structured cultural curricula and co-curricular learning experiences through which students encounter collective histories and interpret their cultural inheritance. Engagement with heritage-related courses, campus activities, and experiential learning can facilitate meaning-making and strengthen self-definition as a member of a broader cultural community. Prior work on cultural socialization and identity formation suggests that greater exposure to cultural traditions and collective narratives is associated with stronger cultural identification. Accordingly, cultural heritage education is expected to positively predict cultural identity.

H1: *Cultural heritage education positively predicts college students' cultural identity*.

#### 2.1.2 Cultural heritage education and cultural pride

Beyond cognitive identification, cultural heritage education may also elicit affective attachment toward one's cultural community. Cultural pride refers to positive emotional evaluations of cultural heritage, such as admiration, respect, and a sense of collective value. Educational experiences that highlight cultural achievements, historical continuity, and symbolic meaning can evoke pride and deepen students' emotional investment in cultural heritage. Such affective responses can motivate individuals to value, preserve, and represent their cultural community in prosocial ways. Therefore, cultural heritage education is expected to positively predict cultural pride.

H2: *Cultural heritage education positively predicts college students' cultural pride*.

#### 2.1.3 Cultural identity as a cognitive pathway to civic engagement

Cultural identity provides a cognitive foundation for value internalization and civic-related behavior. Students who strongly identify with their cultural community are more likely to endorse collective goals, perceive public participation as consistent with their self-concept, and interpret civic action as meaningful for the community to which they belong. Social identity perspectives further suggest that stronger group identification can motivate prosocial behaviors that benefit the broader collective. Empirical research has also associated cultural identification with greater public-mindedness and civic participation. Thus, cultural identity is expected to positively predict civic engagement among university students.

H3: *Cultural identity positively predicts college students' civic engagement*.

#### 2.1.4 Cultural pride as an affective pathway to civic engagement

Cultural pride functions as an affective motivator that can energize civic behavior. Positive emotions associated with cultural heritage may broaden individuals' orientations toward the common good and increase willingness to contribute to collective well-being. Students who experience pride in cultural heritage may feel motivated to participate in civic and community-oriented activities that reflect and sustain valued cultural meanings. Accordingly, cultural pride is expected to positively predict civic engagement.

H4: *Cultural pride positively predicts college students' civic engagement*.

#### 2.1.5 Parallel mediation through cultural identity and cultural pride

Value-socialization theory posits that educational experiences influence behavioral outcomes by shaping internal orientations that guide action. In the present model, cultural heritage education is expected to relate to civic engagement through two parallel mechanisms: a cognitive route via cultural identity and an affective route via cultural pride. Accordingly, CHE is expected to exert two simultaneous indirect effects on CE, one through CI and the other through CP. In addition, consistent with a pragmatic value-socialization perspective, the model acknowledges that a remaining direct association between CHE and CE may reflect additional unmeasured pathways; therefore, the empirical analyses test both a fully mediated baseline and a partial-mediation specification.

H5: *Cultural identity and cultural pride mediate the relationship between cultural heritage education and civic engagement through two parallel indirect effects*.

#### 2.1.6 Residual direct association between cultural heritage education and civic engagement

In addition to the hypothesized indirect pathways through cultural identity and cultural pride, we further examined whether a residual direct association between cultural heritage education (CHE) and civic engagement (CE) remained after accounting for these mediators. Specifically, we compared a partial-mediation model, in which the direct path from CHE to CE was freely estimated, with a fully mediated model in which this path was constrained to zero. This comparison allowed us to evaluate whether cultural heritage education exerted an additional influence on civic engagement beyond the proposed cognitive and affective mechanisms. The results of this model comparison are reported in the following section.

## 3 Research methods

This study employed a cross-sectional survey design to examine the associations among cultural heritage education (CHE), cultural identity (CI), cultural pride (CP), and civic engagement (CE) among Chinese university students. Drawing on value-socialization theory, the hypothesized model specified two parallel psychological pathways (CI and CP) linking CHE to CE. We evaluated both a fully mediated baseline model (Model A; constraining CHE → CE to zero) and a partial-mediation model (Model B; freely estimating CHE → CE) to test whether allowing a residual direct path improved model fit and to benchmark the hypothesized mediation structure. Model B was treated as the primary model for inference if it showed significantly better fit than Model A based on the chi-square difference test for nested models and information criteria (AIC).

### 3.1 Participants and procedure

Participants were Chinese undergraduate students recruited via online announcements distributed through university learning platforms and student communication channels. Eligibility criteria required current full-time undergraduate enrollment. Participation was voluntary, anonymous, and uncompensated.

Before accessing the questionnaire, participants viewed an online informed consent statement describing the study purpose, confidentiality protections, and their right to withdraw at any time without penalty. Only participants who provided electronic informed consent were permitted to proceed to the survey. Data were collected using Wenjuanxing, a widely used online survey platform in China. Participants were instructed to complete the questionnaire independently and respond as honestly as possible. The average completion time was approximately 15 min.

A pilot study was conducted prior to the main data collection to assess item clarity and preliminary psychometric properties. After data screening, 59 valid responses were retained for pilot analyses. For the main study, a total of 744 valid questionnaires were obtained after excluding responses with unrealistically short completion times, evident patterned responding, or other indicators of insufficient effort.

The analytic sample consisted exclusively of undergraduate students, resulting in a relatively restricted age range with limited variance. Given this homogeneity and the study's theoretical focus on psychological value-internalization mechanisms rather than demographic prediction, demographic variables such as age were not included as exogenous controls in the structural model. This modeling decision was intended to maintain conceptual parsimony and to align the analysis with the primary research objective of examining mediation processes linking cultural heritage education to civic engagement through cultural identity and cultural pride.

The study employed an anonymous, non-interventional online questionnaire and involved no deception. No personally identifiable information (e.g., names, student identification numbers, or contact details) was collected. All responses were analyzed in aggregated form to protect confidentiality. The study targeted adult undergraduate participants and posed no more than minimal risk. Data were stored securely and used solely for research purposes. All participants provided informed consent prior to participation. The study involved anonymous, self-administered questionnaires and posed minimal risk to participants, and ethical procedures were conducted in accordance with institutional guidelines.

### 3.2 Measures

All constructs were assessed using self-report items administered in Chinese. The questionnaire included four variables: cultural heritage education (CHE; 5 items), cultural identity (CI; 4 items), cultural pride (CP; 4 items), and civic engagement (CE; 4 items). Items were rated on a 5-point Likert scale (1 = *strongly disagree* to 5 = *strongly agree*). Scale scores were computed as the mean of the items for each construct, with higher scores indicating higher levels of the corresponding variable. Detailed information regarding item sources, adaptations, and theoretical rationales for all measurement scales is provided in Supplementary Table S1.

#### 3.2.1 Cultural heritage education

CHE assessed students' perceived exposure to and engagement with cultural-heritage learning opportunities in the university context. The five items were developed for the present study to reflect common forms of heritage education in Chinese higher education, covering heritage-related coursework, availability of heritage activities, students' proactive participation, perceived campus learning atmosphere, and the perceived importance of heritage learning within one's university experience. Item wording was refined based on feedback from a pilot test (*N* = 59) to improve clarity and contextual appropriateness while preserving the intended construct meaning. The full wording of all measurement items (Chinese originals with English translations) is provided in the Supplementary Appendix A to ensure transparency and replicability (Valencia Arnica et al., 2023; Monteagudo-Fernández et al., 2021).

#### 3.2.2 Cultural identity

CI captured students' cognitive identification with and sense of belonging to Chinese culture (e.g., cultural belonging, perceived relevance of cultural traditions and history to one's identity, heritage as part of “who I am,” and the importance of being part of Chinese culture). Item content was informed by widely used work on ethnic/cultural identity and social identity-based self-definition (Phinney, 1992; Umaña-Taylor et al., 2014; Leach et al., 2008; Schwartz et al., 2011).

#### 3.2.3 Cultural pride

CP reflected positive affective evaluation and emotional attachment toward Chinese cultural heritage (e.g., feeling proud of one's connection with traditional culture, increased pride after learning about heritage, feeling honored when Chinese culture is displayed or disseminated, and pride in the long history and achievements of Chinese civilization). Item content was informed by prior research on group-based emotions and positive evaluation of social identity (Smith et al., 2007; Leach et al., 2007; Luhtanen and Crocker, 1992; Tracy and Robins, 2007).

#### 3.2.4 Civic engagement

CE assessed students' intention and self-reported tendency to participate in civic- and community-oriented activities (e.g., willingness to join volunteering/community service, interest in activities that promote social development, willingness to invest time/effort to improve the community, and future intention to serve society more). Item content was informed by commonly used measures and conceptualizations of civic engagement in youth and higher education research (Ehrlich, 2000; Zaff et al., 2010; Doolittle and Faul, 2013).

#### 3.2.5 Content validity and pilot testing

Items were written and administered in Chinese. Prior to the main study, a pilot test (*N* = 59) was conducted to evaluate clarity and contextual appropriateness. Minor wording refinements were made based on participant feedback while preserving the intended construct meanings.

#### 3.2.6 Psychometric evaluation

Reliability and validity were evaluated in the main sample using confirmatory factor analysis (CFA). Internal consistency (Cronbach's α), composite reliability (CR), and average variance extracted (AVE) were computed for each construct, and discriminant validity was examined using the Fornell–Larcker criterion (see Section 4).

### 3.3 Data analysis

Given that the primary aim of this study was to examine a mechanism-oriented value-socialization model involving multiple latent constructs and parallel mediation pathways, structural equation modeling (SEM) was selected as the primary analytic strategy. SEM is particularly suitable for simultaneously estimating relationships among latent variables, accounting for measurement error, and testing complex mediation structures within a theoretically specified model. All statistical analyses were conducted using SPSS Statistics 27.0, IBM Corp., Armonk, NY, United States and AMOS (version 31.0).

First, descriptive statistics and Pearson correlation analyses were performed in SPSS to examine preliminary associations among cultural heritage education, cultural identity, cultural pride, and civic engagement.

Potential common method bias was assessed using two complementary approaches. In SPSS, Harman's single-factor test was conducted via exploratory factor analysis (principal axis factoring), including all measurement items without rotation. In AMOS, a single-factor confirmatory factor analysis (CFA) model was also estimated by loading all items onto one latent factor. Model fit indices were examined to assess whether a single common factor could adequately account for the covariance among the observed measures.

SEM analyses followed a two-step modeling procedure. First, a CFA was conducted to evaluate the measurement model and assess construct validity for all latent variables. Second, the hypothesized structural model was tested to examine the relationships among cultural heritage education, cultural identity, cultural pride, and civic engagement.

To examine whether cultural identity and cultural pride fully or partially mediated the association between cultural heritage education (CHE) and civic engagement (CE), two theoretically specified structural models were estimated and compared. A fully mediated baseline model (Model A) constrained the direct path from CHE to CE to zero, reflecting a strict value-socialization assumption. A partial mediation model (Model B) additionally included a direct CHE → CE path to test whether any residual association remained after accounting for the two mediators. The two nested models were compared using a chi-square difference test and Akaike Information Criterion (AIC) values to evaluate relative model fit and robustness.

Overall model fit was evaluated using multiple indices, including the chi-square statistic, the comparative fit index (CFI), the Tucker–Lewis index (TLI), and the root mean square error of approximation (RMSEA). Indirect effects were tested using a bias-corrected bootstrap procedure with 5,000 resamples. All statistical tests were two-tailed, with a significance level set at 0.05.

## 4 Results

### 4.1 Sample characteristics

The final sample consisted of 744 Chinese undergraduate students. As shown in Table 1, 42.9% of participants were male (*n* = 319) and 57.1% were female (*n* = 425). The sample was relatively evenly distributed across grade levels (Year 1–Year 4). Participants represented a broad range of academic disciplines, with comparable proportions from Humanities/History/Philosophy (24.5%), Economics/Management/Law (25.7%), Arts/Education (24.1%), and Science/Engineering (25.8%).

**Table 1:** Sample characteristics (*N* = 744).

**Characteristic**	**n**	**%**
**Gender**		
Male	319	42.9
Female	425	57.1
**Grade**		
Freshman (Year 1)	172	23.1
Sophomore (Year 2)	182	24.5
Junior (Year 3)	202	27.2
Senior (Year 4)	188	25.3
**Major/discipline**		
Humanities/history/ philosophy	182	24.5
Economics/management/ law	191	25.7
Arts/education	179	24.1
Science/engineering	192	25.8

Percentages are based on the valid sample (*N* = 744) and may not sum to 100 due to rounding. The pilot sample (*N* = 59) was used for instrument checks and is described in the Section 3.

### 4.2 Descriptive statistics, correlations, and discriminant validity

Table 2 presents descriptive statistics and correlations among the study variables. Participants reported relatively high levels of CHE (*M* = 4.17, *SD* = 0.75), CI (*M* = 4.22, *SD* = 0.73), CP (*M* = 4.23, *SD* = 0.74), and CE (*M* = 4.21, *SD* = 0.73). CHE was positively correlated with CI (*r* = 0.28, *p* < 0.001), CP (*r* = 0.31, *p* < 0.001), and CE (*r* = 0.28, *p* < 0.001). CI and CP were also positively correlated with CE (*r* = 0.30 and *r* = 0.29, respectively, both *p* < 0.001). Discriminant validity was supported by the Fornell–Larcker criterion, as the square roots of AVE (diagonal) exceeded the inter-construct correlations.

**Table 2:** Descriptive statistics, correlations, and Fornell–Larcker discriminant validity (*N* = 744).

**Variable**	**Mean**	**SD**	**1**	**2**	**3**	**4**
1. CHE	4.17	0.75	**0.741**			
2. CI	4.22	0.73	0.28^***^	**0.740**		
3. CP	4.23	0.74	0.31^***^	0.33^***^	**0.755**	
4. CE	4.21	0.73	0.28^***^	0.30^***^	0.29^***^	**0.733**

Pearson correlations are reported (two-tailed). Bold diagonal values are the square roots of the average variance extracted ($\sqrt{\text{AVE}}$) for each construct (Fornell–Larcker criterion). Discriminant validity is supported when each $\sqrt{\text{AVE}}$ exceeds the correlations with other constructs. CHE, cultural heritage education; CI, cultural identity; CP, cultural pride; CE, civic engagement. ^*^p < 0.05, ^**^p < 0.01, ^***^p < 0.001.

### 4.3 Common method bias assessment

Common method bias was assessed using Harman's single-factor test and a single-factor CFA. For Harman's test, an unrotated exploratory factor analysis (principal axis factoring) was conducted including all measurement items. Four factors with eigenvalues greater than 1 emerged, and the first factor accounted for 32.45% of the total variance, below the commonly used 40% criterion. In addition, the single-factor CFA model (all items loading on one latent factor) demonstrated very poor fit, χ^2^(119) = 3, 326.62, χ^2^/*df* = 27.96, CFI = 0.457, TLI = 0.379, and RMSEA = 0.190 (90% CI [0.185, 0.196]). Taken together, the results indicate that the covariance among the measures is unlikely to be dominated by a single common-method factor; however, consistent with best practice for cross-sectional self-report designs, common method effects cannot be fully ruled out.

### 4.4 Measurement model

A confirmatory factor analysis (CFA) was conducted to evaluate the four-factor measurement model comprising cultural heritage education (CHE), cultural identity (CI), cultural pride (CP), and civic engagement (CE). The model showed good fit to the data, χ^2^(113) = 329.416, χ^2^/*df* = 2.915, CFI = 0.963, TLI = 0.956, and RMSEA = 0.051 (90% CI [0.044, 0.057], PCLOSE = 0.410), with acceptable absolute fit indices (GFI = 0.947, AGFI = 0.928, RMR = 0.028).

All standardized factor loadings were substantial and exceeded 0.70 (Table 3), supporting indicator reliability. Reliability and convergent validity were further supported (Table 4): Cronbach's α values ranged from 0.844 to 0.875, CR ranged from 0.823 to 0.859, and AVE ranged from 0.538 to 0.570, meeting recommended thresholds for internal consistency (Cronbach's α>0.70, CR >0.70) and convergent validity (AVE >0.50).

**Table 3:** Standardized factor loadings for the measurement model (*N* = 744).

**Construct**	**Item**	**Loading (λ)**	**SMC**
CHE	CHE1	0.799	0.638
	CHE2	0.736	0.542
	CHE3	0.805	0.648
	CHE4	0.738	0.545
	CHE5	0.742	0.551
CI	CI1	0.724	0.524
	CI2	0.768	0.590
	CI3	0.760	0.578
	CI4	0.781	0.610
CP	CP1	0.763	0.582
	CP2	0.771	0.595
	CP3	0.809	0.654
	CP4	0.780	0.608
CE	CE1	0.792	0.627
	CE2	0.711	0.506
	CE3	0.776	0.602
	CE4	0.756	0.572

λ, standardized factor loading; SMC, squared multiple correlation.

**Table 4:** Reliability and convergent validity of the measurement model (*N* = 744).

**Construct**	**Items**	**Cronbach's α**	**CR**	**AVE**
CHE (Cultural heritage education)	5	0.875	0.859	0.549
CI (Cultural identity)	4	0.844	0.828	0.547
CP (Cultural pride)	4	0.862	0.842	0.570
CE (Civic engagement)	4	0.844	0.823	0.538

CR, composite reliability; AVE, average variance extracted. All standardized factor loadings were significant and exceeded 0.70.

### 4.5 Structural model and hypothesis testing

**Figure 2:**
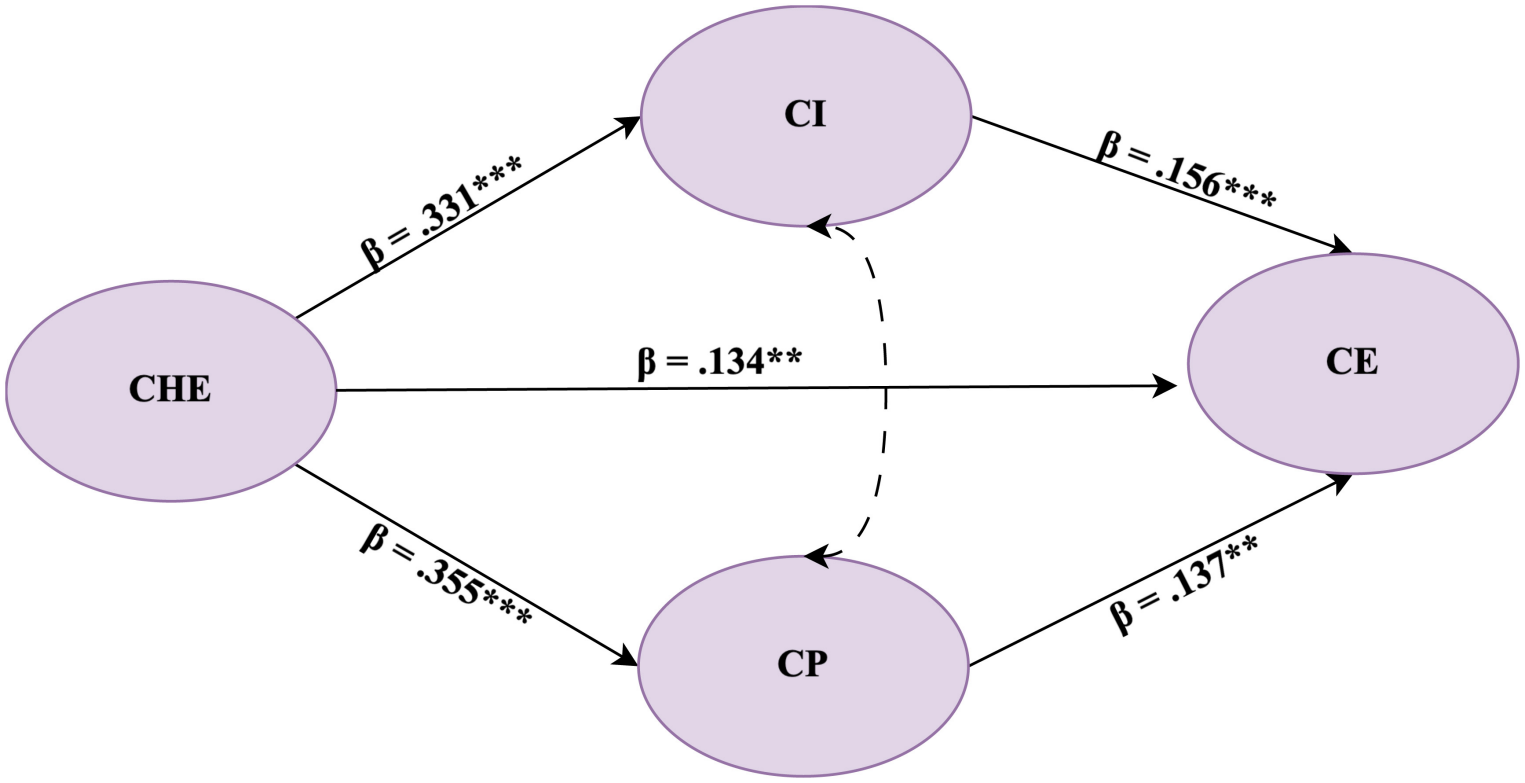
Standardized structural model (Model B) with parallel mediation. Cultural heritage education (CHE) positively predicts cultural identity (CI) and cultural pride (CP), which in turn positively predict civic engagement (CE). A residual direct path from CHE to CE is retained to test partial mediation. Values on the arrows are standardized path coefficients (β). The curved double-headed arrow denotes the covariance between the disturbances of CI and CP. ^*^p < 0.05, ^**^p < 0.01, ^***^p < 0.001.

Structural equation modeling (SEM) was conducted to test the hypothesized relationships among cultural heritage education, cultural identity, cultural pride, and civic engagement. Based on the latest model comparison results, the primary model was specified as a partial-mediation model (Model B) that included the direct path CHE → CE. Model B demonstrated an acceptable fit to the data, χ^2^/*df* = 4.33, CFI = 0.936, TLI = 0.923, and RMSEA = 0.067. Figure 2 presents the standardized structural model for the primary partial-mediation specification (Model B). For clarity, the figure displays only the latent constructs and the structural paths among them. As shown in the model, CHE positively predicts CI and CP, and both mediators are positively associated with CE. In addition, a residual direct path from CHE to CE is retained to test partial mediation, and the residuals of CI and CP are allowed to correlate to account for shared unexplained variance. Detailed estimates and explained variance for each endogenous construct are reported in Table 5.

**Table 5:** Structural model results and explained variance (Model B; *N* = 744).

**Hypothesis**	**Path**	**B**	**SE**	**C.R**.	***p***	**β**	***R*^2^ (DV)**
H1	CHE → CI	0.269	0.035	7.604	<0.001	0.331	0.109
H2	CHE → CP	0.307	0.037	8.303	<0.001	0.355	0.126
H3	CI → CE	0.270	0.080	3.363	<0.001	0.156	0.105
H4	CP → CE	0.224	0.075	2.970	0.003	0.137	0.105
—	CHE → CE (direct)	0.190	0.063	3.030	0.002	0.134	0.105

CHE, cultural heritage education; CI, cultural identity; CP, cultural pride; CE, civic engagement. *B*, unstandardized estimate; SE, standard error; C.R., critical ratio. Standardized coefficients (β) are reported for effect size interpretation. *R*^2^ indicates the squared multiple correlation (explained variance) for the dependent variable (DV) of each equation (CI = 0.109; CP = 0.126; CE = 0.105). The CHE → CE path is included in Model B to test partial mediation and is not tied to H1–H4.

As shown in Table 5, cultural heritage education positively predicted cultural identity (β = 0.331, *p* < 0.001) and cultural pride (β = 0.355, *p* < 0.001), supporting H1 and H2. In turn, cultural identity (β = 0.156, *p* < 0.001) and cultural pride (β = 0.137, *p* = 0.003) were positively associated with civic engagement, supporting H3 and H4. Importantly, the direct effect of cultural heritage education on civic engagement also remained statistically significant (β = 0.134, *p* = 0.002), indicating partial mediation rather than a fully mediated relationship. With respect to explained variance, CHE accounted for 10.9% of the variance in cultural identity and 12.6% in cultural pride, and the full model explained 10.5% of the variance in civic engagement.

### 4.6 Alternative model comparison

Accordingly, Model B was retained as the primary model for hypothesis testing, and Model A served as a nested baseline for comparison. To benchmark the primary partial-mediation model, a fully mediated baseline model (Model A) was also estimated by constraining the direct path CHE → CE to zero. Model A showed a comparable but slightly poorer fit than Model B (Model A: χ^2^/*df* = 4.38, CFI = 0.934, TLI = 0.922, RMSEA = 0.067; Model B: χ^2^/*df* = 4.33, CFI = 0.936, TLI = 0.923, RMSEA = 0.067). A chi-square difference test indicated that freeing the CHE → CE path significantly improved model fit, Δχ^2^(1) = 10.00, *p* = 0.002. Consistent with this improvement, Model B also yielded a lower AIC (571.97 vs. 579.97) and higher explained variance in civic engagement ($R_{CE}^{2}=0.105$ vs. 0.092). Therefore, Model B was retained as the primary model for inference.

### 4.7 Parallel mediation analysis

Bootstrapping analyses with 5,000 resamples were conducted to examine the indirect effects of cultural heritage education on civic engagement through cultural identity and cultural pride. As reported in Table 6, the indirect effect via cultural identity was statistically significant (indCI = 0.073, BC 95% CI [0.040, 0.118], *p* < 0.001), and the indirect effect via cultural pride was also statistically significant (indCP = 0.069, BC 95% CI [0.036, 0.114], *p* < 0.001). The total indirect effect was significant (indTotal = 0.141, BC 95% CI [0.090, 0.203], *p* < 0.001), indicating that cultural identity and cultural pride function as parallel mediators linking cultural heritage education to civic engagement. In addition, the direct effect remained statistically significant (direct = 0.190, BC 95% CI [0.114, 0.277], *p* < 0.001), consistent with partial mediation. The total effect was also significant (total = 0.331, BC 95% CI [0.225, 0.444], *p* < 0.001).

**Table 6:** Bootstrap effects (bias-corrected) for the partial mediation model (Model B; *N* = 744).

**Effect**	**Estimate**	**BC 95% CI**	***p***
Indirect: CHE → CI → CE (indCI)	0.073	[0.040, 0.118]	<0.001
Indirect: CHE → CP → CE (indCP)	0.069	[0.036, 0.114]	<0.001
Total indirect effect (indTotal)	0.141	[0.090, 0.203]	<0.001
Direct: CHE → CE (direct)	0.190	[0.114, 0.277]	<0.001
Total effect (total)	0.331	[0.225, 0.444]	<0.001

Bias-corrected (BC) percentile bootstrap confidence intervals were obtained using 5,000 resamples in AMOS. CHE, cultural heritage education; CI, cultural identity; CP, cultural pride; CE, civic engagement. Effects are considered statistically significant when their confidence intervals do not include zero.

## 5 Discussion

Grounded in value-socialization theory, this study examined how cultural heritage education (CHE) is associated with civic engagement (CE) among Chinese university students via two parallel psychological mechanisms: cultural identity (CI) and cultural pride (CP). Using SEM, the results supported a mechanism-oriented account in which CHE was positively related to both CI and CP, and each of these orientations was positively associated with CE. Importantly, the preferred structural specification was a *partial-mediation* model (Model B): beyond the two indirect pathways, CHE retained a statistically significant direct association with CE. Given the cross-sectional, self-report design, these findings should be interpreted as evidence of associations consistent with the proposed model, rather than as definitive causal effects.

### 5.1 Main findings

Three findings merit emphasis.

First, CHE was positively associated with both CI and CP. Students reporting greater exposure to heritage-related learning experiences also reported stronger cultural identity (β = 0.331, *p* < 0.001) and greater cultural pride (β = 0.355, *p* < 0.001). This finding aligns with value-socialization theory in that educational experiences can shape both cognitive self-definition (identity) and affective attachment (pride) toward cultural heritage.

Second, both CI and CP were positively linked to CE. Cultural identity (β = 0.156, *p* < 0.001) and cultural pride (β = 0.137, *p* = 0.003) each contributed unique variance to civic engagement in the same structural model. Although modest in magnitude, these effects are meaningful in a large undergraduate sample and indicate that civic participation is associated not only with heritage exposure per se, but also with how students incorporate cultural meanings into their self-concept and emotional evaluations.

Third, the mediation evidence supported the proposed parallel mechanisms while also indicating partial mediation. Bias-corrected bootstrap tests showed significant indirect effects through both CI (indCI = 0.073, BC 95% CI [0.040, 0.118], *p* < 0.001) and CP (indCP = 0.069, BC 95% CI [0.036, 0.114], *p* < 0.001), with a significant total indirect effect (indTotal = 0.141, BC 95% CI [0.090, 0.203], *p* < 0.001). At the same time, the direct effect remained significant (direct = 0.190, BC 95% CI [.114, 0.277], *p* < 0.001), and the total effect was also significant (total = 0.331, BC 95% CI [.225, 0.444], *p* < 0.001). Together, these results indicate that CI and CP function as reliable parallel mediators, but they do not exhaust the linkage between CHE and civic engagement.

### 5.2 Theoretical implications

A key theoretical implication of the present findings lies in the conceptual differentiation between cultural identity and cultural pride as two distinct yet complementary value-socialization pathways. In line with the proposed framework, cultural identity is understood as a cognitive process of self-definition, reflecting individuals' perceived cultural belonging and incorporation of cultural meanings into the self-concept. By contrast, cultural pride is explicitly conceptualized as an affective evaluative orientation toward cultural heritage, capturing positive emotional appraisal and attachment rather than cognitive self-categorization.

Importantly, cultural pride is not treated as a cognitive component or sub-dimension of cultural identity. Instead, cultural identity and cultural pride are modeled as parallel mediators that operate through different psychological mechanisms. Whereas cultural identity addresses the question of “who I am” in relation to cultural heritage, cultural pride reflects “how I feel about what I belong to.” By modeling these constructs as parallel rather than hierarchical processes, the present study highlights that educational experiences may foster civic engagement not only by shaping students' cultural self-definitions, but also by cultivating positive emotional attachment and evaluative appreciation toward cultural heritage. In this sense, the present findings empirically operationalize value socialization as a dual-process mechanism involving both cognitive internalization and affective evaluation within the context of higher education.

This study contributes to the literature in several ways. First, it refines value-socialization accounts by identifying two complementary psychological routes through which cultural heritage education relates to civic engagement. Rather than treating cultural heritage education as a diffuse form of cultural exposure, the proposed model specifies a cognitive pathway (cultural identity) and an affective pathway (cultural pride), both of which independently link heritage education to civic engagement. Providing this mechanism-level distinction enhances theoretical precision regarding how educational inputs translate into civic outcomes.

Second, the finding of partial mediation offers a theoretically realistic extension of socialization models. Many value-socialization processes operate through multiple channels, and it is plausible that cultural heritage education also influences civic engagement through additional mechanisms not captured in the present model, such as civic efficacy, moral obligation, social norms, or increased access to civic opportunities within universities and surrounding communities. Retaining a direct path from cultural heritage education to civic engagement acknowledges these possibilities and avoids over-attribution to any single explanatory mechanism.

Third, the results help clarify the joint roles of identity and emotion in civic development models. Demonstrating simultaneous contributions of cognitive identification (cultural identity) and affective evaluation (cultural pride) bridges identity-based perspectives on civic behavior with emotion-based accounts of prosocial motivation. In educational contexts where heritage education aims to foster public-minded orientations and civic participation, both meaning internalization and positive emotional attachment appear to play important roles. Future theory-building may further examine the conditions under which one pathway becomes more salient than the other, such as variations in perceived authenticity of heritage experiences, campus cultural climate, or students' prior engagement with cultural heritage.

### 5.3 Practical implications

Several actionable implications follow for universities and educators.

First, heritage education may be most effective when it is designed to cultivate both identity and pride, rather than focusing narrowly on factual knowledge. To strengthen cultural identity, curricular designs can incorporate reflective and meaning-making components (e.g., guided discussion, narrative analysis, reflective writing) that connect heritage content to students' self-definition and perceived cultural membership.

Second, to foster cultural pride, heritage education should include emotionally engaging and experiential elements. Field visits, hands-on workshops, community-based documentation projects, and collaboration with cultural practitioners can enhance emotional connection and evaluative appreciation, especially when the activities are perceived as authentic and personally relevant.

Third, because civic engagement is ultimately behavioral, universities should translate heritage learning into structured opportunities for civic action. “Heritage-to-civic” pathways can be built through service-learning, volunteering, community education, public cultural events, or project-based courses that require civic contribution. Such designs help ensure that identity and pride are not only internalized, but also expressed through sustained participation.

### 5.4 Limitations

Several limitations should be considered when interpreting these findings.

First, the cross-sectional design precludes strong causal inference and temporal ordering. It is plausible that civically engaged students are more likely to participate in, attend to, or positively report heritage-related learning experiences. Longitudinal, panel, and intervention designs are needed to test directional effects and change processes.

Second, all constructs were assessed via self-report at a single time point, which may inflate associations due to common method variance and social desirability. Future work should incorporate multi-source data and behavioral indicators of civic engagement (e.g., verified volunteering hours, participation records) and separate measurement occasions for predictors, mediators, and outcomes.

Third, the sample consisted of Chinese undergraduates recruited online, which may limit generalizability to other age groups, educational systems, or cultural contexts. Replication across regions, institutional types, and countries would help establish boundary conditions.

Finally, the explained variance in civic engagement was modest ($R_{CE}^{2}=0.105$), which is common for broad civic outcomes but indicates that additional predictors and contextual factors remain important. Civic engagement is a distal and multifaceted outcome; therefore, modest explained variance is common and theoretically expected in cross-sectional psychosocial models. Integrating variables such as civic efficacy, perceived social norms, sense of community, and the perceived authenticity/quality of heritage education may improve explanatory power and sharpen theoretical inference.

### 5.5 Future directions and summary

Future research can extend this work in several directions. First, longitudinal and quasi-experimental studies are needed to test whether CHE predicts changes in CI, CP, and civic engagement over time and to identify which instructional components (e.g., experiential learning, reflection, and community interaction) are most effective.

Second, multi-method approaches should be adopted. Combining self-report measures with behavioral/administrative indicators and using time-lagged measurement can reduce method bias and strengthen mediation inference.

Third, boundary conditions and moderators warrant examination. Prior heritage involvement, perceived authenticity, campus cultural climate, and socioeconomic background may influence whether identity-based or pride-based pathways are more salient. Expanding the model to include additional mechanisms (e.g., civic efficacy, moral identity, and perceived civic norms) may also provide a more comprehensive account of how heritage education connects to civic participation.

In sum, this study provides evidence for a mechanism-oriented value-socialization model in which cultural heritage education is positively associated with civic engagement through two parallel pathways—cultural identity and cultural pride—while also retaining a significant direct association. The findings suggest that heritage education may support civic engagement not only through knowledge and internalized cultural orientations, but also through additional, potentially contextual routes that deserve further investigation.

## 6 Conclusion

This study advanced a value-socialization framework to clarify how cultural heritage education may be associated with civic engagement among Chinese university students. Using a partial-mediation structural model, the findings indicate that cultural heritage education is positively associated with civic engagement through two concurrent psychological pathways: a cognitive route via cultural identity and an affective route via cultural pride. Both indirect effects were significant, and a remaining direct association between heritage education and civic engagement was also observed, suggesting that identity and pride are important—but not exclusive—channels through which heritage learning relates to civic participation.

These results underscore that cultural heritage education in higher education can function as more than knowledge transmission. When cultural programs help students internalize cultural meanings and experience positive emotional connection, they may be more likely to engage in civic and community-oriented activities. Practically, universities may strengthen the civic value of heritage education by designing learning experiences that combine reflection-based identity work, emotionally engaging and authentic cultural encounters, and clear “heritage-to-civic” opportunities such as service-learning, volunteering, and community co-creation.

Overall, this study provides empirically grounded support for a mechanism-oriented account of the cultural heritage education–civic engagement link and offers actionable implications for integrating cultural learning with civic development in contemporary China.

## Data Availability

The raw data supporting the conclusions of this article will be made available by the authors, without undue reservation.
