# Supplementary material for: Cultural heritage education and civic engagement: a value-socialization model among Chinese university students
Source: Front Psychol. 2026 Feb 26;17:1779128. doi: 10.3389/fpsyg.2026.1779128 (PMC12979467; doi:10.3389/fpsyg.2026.1779128)
Supplement: Supplementary file 1 [file Supplementary_file_1.docx]

**Cultural Heritage Education and Civic Engagement: A Value-Socialization Model among Chinese University Students**

**Xu Yu^1，*^**

Hangzhou Polytechnic University, School of Animation and Games, Xueyuan Street, Hangzhou, 310000, Zhejiang, China.

2017010022@hzvtc.edu.cn

**Supplementary Material**

**Appendix A. Measurement Instruments**

**This appendix provides the full list of measurement items used in the present study. All items were administered in Chinese. English translations are provided for transparency and replication purposes. All items were rated on a five-point Likert scale ranging from 1 (*strongly disagree*) to 5 (*strongly agree*). Items within each construct were averaged to create composite scale scores, with higher scores indicating higher levels of the corresponding construct.**

**A1. Cultural Heritage Education (CHE)**

***(Developed for the present study)***

**Response scale: 1 = strongly disagree, 5 = strongly agree**

1. **CHE1
   我修读过与文化遗产或传统文化相关的课程或模块。
   *I have taken courses or modules related to cultural heritage or traditional culture.***
2. **CHE2
   学校提供了丰富的文化遗产相关活动（如讲座、非遗体验、文化节等）。
   *My university provides abundant cultural heritage–related activities (e.g., lectures, intangible cultural heritage experiences, cultural festivals).***
3. **CHE3
   我曾主动参加文化遗产相关的校园活动。
   *I have actively participated in cultural heritage–related activities on campus.***
4. **CHE4
   我认为学校营造了良好的文化遗产学习氛围。
   *I believe my university has created a positive learning atmosphere for cultural heritage.***
5. **CHE5
   文化遗产相关内容是我大学学习经验的重要组成部分。
   *Cultural heritage–related learning is an important part of my university experience.***

**A2. Cultural Identity (CI)**

**Response scale: 1 = strongly disagree, 5 = strongly agree**

1. **CI1
   我对中华文化具有强烈的归属感。
   *I have a strong sense of belonging to Chinese culture.***
2. **CI2
   我认为文化传统与历史与我的身份认同密切相关。
   *I believe that cultural traditions and history are closely related to my sense of identity.***
3. **CI3
   文化遗产是理解“我是谁”的重要组成部分。
   *Cultural heritage is an important part of understanding who I am.***
4. **CI4
   成为中华文化的一部分对我来说非常重要。
   *Being part of Chinese culture is very important to me.***

**A3. Cultural Pride (CP)**

**Response scale: 1 = strongly disagree, 5 = strongly agree**

1. **CP1
   我为自己与中华传统文化有联系而感到自豪。
   *I feel proud to be connected with traditional Chinese culture.***
2. **CP2
   学习文化遗产让我对自己的文化背景感到更加自豪。
   *Learning about cultural heritage makes me feel more proud of my cultural background.***
3. **CP3
   当中华文化被展示或传播时，我会感到荣誉感。
   *I feel a sense of honor when Chinese culture is displayed or promoted.***
4. **CP4
   我为中华文明悠久的历史和成就而感到自豪。
   *I am proud of the long history and achievements of Chinese civilization.***

**A4. Civic Engagement (CE)**

**Response scale: 1 = strongly disagree, 5 = strongly agree**

1. **CE1
   我愿意参与志愿服务或社区公益活动。
   *I am willing to participate in volunteering or community service.***
2. **CE2
   我有兴趣参与促进社会发展的活动。
   *I am interested in participating in activities that promote social development.***
3. **CE3
   我愿意投入时间或精力帮助改善社区。
   *I am willing to invest time or effort to help improve the community.***
4. **CE4
   我希望未来更多参与服务社会的活动。
   *I hope to participate more in activities that serve society in the future.***

**Table S1. Sources and Adaptation of Measurement Items**

| Construct | Item Source | Adaptation and Rationale | Key References |
| --- | --- | --- | --- |
| Cultural Heritage Education (CHE) | Developed for this study | Items were developed to capture common forms of cultural heritage education experiences in Chinese higher education, including coursework exposure, campus activities, and perceived institutional support. Item content was informed by prior cultural heritage education frameworks and contextualized to the Chinese university setting. | Valencia Arnica et al. (2023); Monteagudo-Fernandez et al. (2021) |
| Cultural Identity (CI) | Adapted | Items were adapted from established cultural identity scales to reflect students’ sense of belonging to and identification with Chinese culture. Item wording was modified to emphasize heritage-related identity within an educational context. | Phinney (1992); Schwartz et al. (2011) |
| Cultural Pride (CP) | Adapted | Items were adapted to assess affective evaluations of pride related specifically to cultural heritage and traditional culture. The scale emphasizes emotional appraisal rather than cognitive self-definition. | Smith et al. (2007); Tracy & Robins (2007) |
| Civic Engagement (CE) | Adapted | Items were adapted from higher education civic engagement measures, focusing on students’ intentions and willingness to participate in community and social development activities relevant to the university context. | Ehrlich (2000); Doolittle & Faul (2013) |
